# Electroacupuncture Ameliorates Cognitive Impairment by Inhibiting the JNK Signaling Pathway in a Mouse Model of Alzheimer’s Disease

**DOI:** 10.3389/fnagi.2020.00023

**Published:** 2020-02-06

**Authors:** Yinshan Tang, Anping Xu, Shujun Shao, You Zhou, Bing Xiong, Zhigang Li

**Affiliations:** ^1^Department of Rehabilitation and Traditional Chinese Medicine, The Second Affiliated Hospital of Zhejiang University School of Medicine, Hangzhou, China; ^2^School of Acupuncture, Moxibustion and Tuina, Beijing University of Chinese Medicine, Beijing, China

**Keywords:** electroacupuncture, Alzheimer’s disease, APP/PS1 mice, cognitive impairment, JNK signaling pathway, apoptosis

## Abstract

Electroacupuncture (EA) has become popular for its adjustable strength and frequency and easy quantification in the clinic and has demonstrated therapeutic potential for Alzheimer’s disease (AD). However, the mechanism remains unknown. Abnormally activated c-Jun N-terminal kinase (JNK) has been closely related to the pathological process of AD. The aim of this study was to investigate the effect of EA on cognitive impairment and the role of the JNK signaling pathway in AD model amyloid precursor protein (APP)/presenilin 1 (PS1) mice. The memory and learning ability of each group was assessed using the Morris Water Maze (MWM). Immunofluorescence, immunohistochemistry and Western blot were performed to measure the expression of APP, JNK, phosphorylated (P-)JNK, mitogen-activated protein kinase 4 (MKK4), MKK7, c-Jun and caspase-3 in hippocampal tissue samples in APP/PS1 mice after EA intervention. Obvious cognitive deficits were observed in the AD model APP/PS1 mice in the MWM test and were associated with JNK signaling pathway activation and APP upregulation. Four weeks of EA significantly ameliorated the cognitive impairments and inhibited JNK signaling pathway activation and APP upregulation. Taken together, the findings demonstrated that EA can reverse cognitive deficits and substantially lower the burden of APP in AD model APP/PS1 mice, at least partially through inhibiting the JNK signaling pathway and regulating apoptosis signals. Therefore, EA may offer an effective alternative therapeutic approach for AD.

## Introduction

Alzheimer’s disease (AD), the most common cause of dementia, is a slowly progressive neurodegenerative disease that ultimately leads to impairments in several brain functions, such as learning and memory (McKhann et al., 2011). The patient’s ability to live and socialize is severely impaired. The “World Alzheimer Report 2018” noted that is one new dementia patient every three seconds in the world and that by 2050, there will be 152 million dementia patients worldwide. Therefore, AD has become a significant global social burden (Lozano et al., 2012).

The classic amyloid hypothesis states that the deposition of amyloid β (Aβ) protein (Morris et al., 1996; Pike et al., 2007), which is generated by β-amyloid precursor protein (APP) cleavage, is the major event in AD pathology. The aggregation of Aβ initiates irreversible neurodegeneration, which leads to neuronal cell damage and even death (Emerit et al., 2004). Studies have shown that dying cells in the brains of AD patients, including neurons, are characterized by apoptosis (Bredesen et al., 2006).

c-Jun N-terminal kinases (JNKs) belong to the family of stress-activated protein kinases (Kim and Choi, 2010). The JNK pathway plays an important role in a variety of physiological and pathological processes that are mainly involved in apoptosis. JNK activation has been identified as a key element responsible for the regulation of apoptosis signals. Studies have shown that the early activation of JNK is always accompanied by neuronal apoptosis induced by the deposition of Aβ in the brain, indicating that activation of the JNK signaling pathway is involved in the deposition of Aβ and its induced neurotoxicity (Lagalwar et al., 2006; Tare et al., 2011; Li G. Q. et al., 2018). Therefore, abnormally activated JNK is closely related to the pathological process of AD (Ramin et al., 2011; Yarza et al., 2016).

Despite the development of diagnostic techniques for AD, effective and safe therapeutic interventions remain to be found. Because of its curative effects and few side effects, acupuncture has been practiced in more than 183 countries and areas. Compared with manual acupuncture (MA), electroacupuncture (EA) has become popular for its adjustable strength, frequency and easy quantification in the clinic (Pan et al., 2017; Li S. et al., 2018). Studies have also shown that the effect of acupuncture on the brain is integrated at multiple levels, and EA is progressively being used in more clinical practices (Sun et al., 2016; Wei et al., 2016; Chang et al., 2017). In recent years, acupuncture has been used for treating AD and reported to have effectiveness in improving cognitive function (Jiang et al., 2015; Cao et al., 2017; Ding et al., 2019). However, the possible mechanism of action of acupuncture or EA on cognition in AD patients remains uncertain, limiting the application of these studies to guide clinical practice. The mechanism of action of acupuncture must be explored.

Therefore, in this study, we investigated the effect of EA, focusing on the JNK pathway, to determine whether there is an important function of EA in the regulation of the JNK pathway and JNK-dependent apoptosis in AD and further elucidate the mechanism of the potential therapeutic effects of EA.

## Materials and Methods

### Animals and Experimental Groups

Forty 7-month-old male APP/PS1 mice and ten age- and gender-matched C57BL/6 mice were sourced from Beijing HuaFuKang Biotech [Certificate number: SCXK (Jing) 2014-0004], weighing 30 ± 2 g. The APP/PS1 mice were randomly divided into four groups: the model (AD) group, the model + SP600125 (AD + SP) group, the model + EA (AD + EA) group and the model + EA + SP600125 (AD + SP + EA) group, with ten mice in each group. The C57BL/6 mice served as the normal control (N) group. To avoid outside interference, all mice were housed separately in standard mouse cages under constant temperature (23 ± 2°C) and constant humidity (40–60%), with free access to water and food. The study was conducted in strict accordance with the Animal Ethics Committee of Beijing University of Chinese Medicine. We made every effort to minimize the suffering of the animals during the experimental procedure. This animal experiment has been approved by Experimental Animal Ethics Committee of the Second Affiliated Hospital of Zhejiang University (No. 2015-084).

### Apparatus and Reagents

Reagents and apparatuses are displayed in Table 1.

**TABLE 1:** Apparatus and reagents used in this study.

**Apparatus**	**Source**	**Details**
Disposable sterile acupuncture needle	Beijing Zhongyan Taihe Medical Instrument, Co., Ltd	Model: ZYTH2013030504, specification: 0.25 mm × 13 mm
Electroacupuncture apparatus	Peking University Institute of Science Nerve and Beijing Hua Wei Industrial Development Company	Model: HANS-LH202
Morris water maze	Shanghai Xinruan Information Technology, Co., Ltd	Model: XR-XM101
Image acquisition and analysis software	China Daheng Group, Inc	China Daheng Group, Inc., Beijing Image Vision Technology Branch
SP600125	Sigma-Aldrich, St. Louis, MO, United States	

### Methods of Intervention

**FIGURE 1:**
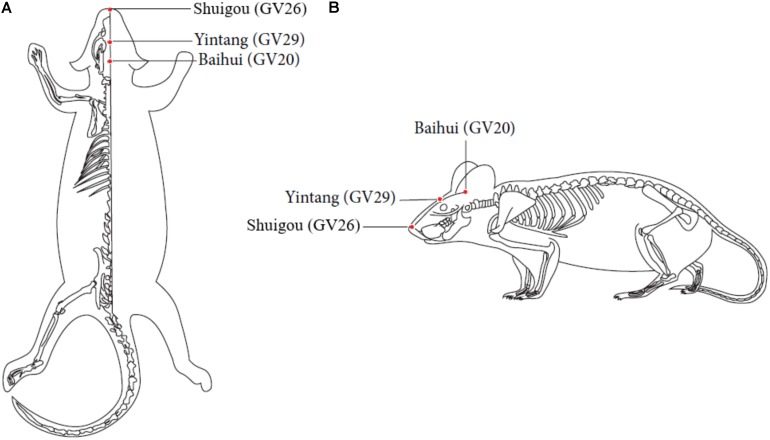
Locations of acupoints we applied in this study. Red points indicate the three governor vessel acupoints respectively: GV20, GV29, and GV26. **(A)** Top views of GV20, GV29, and GV26. **(B)** Locations of GV20, GV29, and GV26.

EA treatment was performed on the AD + EA and AD + SP + EA groups on the acupuncture points Baihui (GV20) (located on the bregma, midpoint of the linking line of mouse ears), Yintang (GV29) (located in the forehead, the middle depression of the two eyebrows at the medial end), and Shuigou (GV26) (located on the face, at the upper one-third of the philtrum) (Figure 1, Tang et al., 2019). Baihui (GV20) and Yintang (GV29) received EA intervention for 20 min, with an intensity of 1 mA and a frequency of 1 Hz, followed by fast pricking of Shuigou (GV26). To immobilize the mice, We have made special bags based on the size of the mice. Ever since the mice crawled inside the bags, two clips would be performed to close the opening back of the bags, which absolutely control and immobilize the mice well. Since the first third of the bags were reticulated, experimental mice could breath properly.

SP600125 was injected intraperitoneally (30 mg/kg) into the AD + SP and AD + SP + EA groups before EA treatment. SP600125 (Sigma-Aldrich, St. Louis, MO, United States) is a JNK pathway blocker and was prepared with 2% dimethyl sulfoxide (DMSO) solvent.

Mice in the N, AD, and AD + SP groups received no EA treatment but were held and bound to ensure an equivalent trial condition. The intervention was performed once every other day for 4 weeks.

### Morris Water Maze (MWM)

Cognitive impairment was assessed using the MWM test, including a training trial and a probe trial to measure memory (D’Hooge and De Deyn, 2001; Vorhees and Williams, 2006). Throughout the training trials, the location of the platform was fixed in the center of the third quadrant (target quadrant) and submerged approximately 1 cm beneath the water surface. The training trial was repeated for five consecutive days. The mice were gently released from the first, second and fourth quadrants, sequentially, in each trial, at an equal distance to the center of the tank. The trial was terminated if the mouse failed to climb onto the platform within 60 s. Spatial acquisition performance was evaluated by assessing the escape latency to reach the platform. A 60-s probe trial was administered 24 h after the last training session. In the probe trial, the platform was removed from the pool, and the mice were placed in the first, second and fourth quadrants, sequentially. The swimming speed, frequency of crossing the platform in the target quadrant (the previous platform location), and percentage of time spent swimming in the third quadrant housing the platform within 60 s were measured by an automated analysis system.

### Sample Collection

Hippocampal sections were isolated and perfused with PBS and 0.2% Triton X-100 and then fixed in 4% paraformaldehyde for 24 h. The left brain sections were rapidly frozen at −50°C and then transversely cut into 5-μm slices.

In addition, seven mice in each group were anesthetized with chloral hydrate, and the hippocampus was collected. The obtained samples were rapidly frozen in liquid nitrogen and stored at −80°C until use.

### Immunohistochemistry

Immunohistochemistry was performed on formalin-fixed, paraffin-embedded right brain hippocampal sections using three 5-μm coronal hippocampal sections per mouse. Anti-mitogen-activated protein kinase kinase 7 (MKK7, 1:100) and c-Jun (60A8) rabbit mAb (1:70) were diluted in PBS, and the sections were incubated at 4°C overnight, washed, and then stained with biotinylated secondary antibody for 10 min. Positive expression was detected by staining the sections with DAB (DAB2031, MXB, China) for 10 min. Microscopy (BX53, Olympus, China) was performed, and images were obtained at 100 × and 400 × magnification. Information on the primary antibodies are displayed in Table 2.

**TABLE 2:** Information on the primary antibodies used in this study.

**Antibody**	**Host**	**Antibody**	**Company**
	**species**	**code**	
Anti-APP antibody [Y188]	Rabbit	ab32136	Abcam, United Kingdom
Anti-JNK1 + JNK2 (phospho T183 + Y185) antibody	Rabbit	ab4821	Abcam, United Kingdom
Anti-JNK	Rabbit	9252S	CST, United States
Anti-MKK7 [EP1455Y]	Rabbit	ab52618	Abcam, United Kingdom
Anti-MKK7(phosphor T275)	Rabbit	ab192592	Abcam, United Kingdom
Anti-MEK4/MKK4	Rabbit	ab131494	Abcam, United Kingdom
Anti-MEK4/MKK4(phospho S80)	Rabbit	ab131353	Abcam, United Kingdom
c-Jun (60A8) rabbit mAb	Rabbit	9165S	CST, United States
Anti-caspase-3	Rabbit	ab13847	Abcam, United Kingdom
Anti-GAPDH	Mouse	ab8245	Abcam, United Kingdom
IgG H&L	Goat	ab150080	Abcam, United Kingdom
Biotinylated secondary antibody	Rabbit	KIT-9706	MXB, China

### Immunofluorescence

Slices were incubated at 4°C with the anti-APP (1:150), anti-MEK4/MKK4 (1:75) and anti-caspase-3 (1:100) antibodies. After permeabilization and blocking overnight, appropriate secondary antibodies (IgG H&L) were used at a dilution of 1:200. After the sections were washed three times with PBS, they were incubated with DAPI (C0065, Solarbio, China) for 10 min, followed by live imaging. The hippocampal images were captured and obtained with a confocal laser microscope (FV1000, Olympus, Japan) at 1000 × magnification. Information on the primary antibodies are displayed in Table 2.

### Western Blot (WB)

The extracted proteins were separated by electrophoresis with 10% SDS-PAGE. The gel was run at 80 V for 20 min and 120 V for 60 min and then transferred onto PVDF membranes at 4°C and 80 V for 1.5 h. The target proteins APP, P-JNK1/2, MKK4, MKK7, c-Jun and caspase-3 were measured by incubation with the primary antibodies against APP (1:2000), JNK1/2 (1:1000), P-JNK1/2 (1:500), MKK4 (1:2000), p-MKK4(1:500), MKK7 (1:1000), p-MKK7(1:500), c-Jun (1:1000) and caspase-3 (1:1000) at 4°C overnight. After the gels were washed three times with TBST, the corresponding secondary antibody was used at a dilution of 1:2000, 1:2000, 1:1000, 1:2000, 1:1000, 1:2000, 1:2000, 1:1000 or 1:2000, respectively, followed by visualization with an ECL kit (mixed with 1:1, PE0010, Solarbio, China). The exposure was completed in a dark room with a chemiluminescence gel imaging system (C600, Azure Biosystems, United States). Antibodies against GAPDH (primary antibody 1:5000 and secondary antibody 1:100000) were used as internal controls. Quantitative results are expressed as a ratio of target proteins to GAPDH and then compared to all groups to measure relative changes. Information on the primary antibodies are displayed in Table 2.

### Statistical Analysis

Statistical analysis was performed using IBM SPSS Statistics 20 software. Two-way ANOVA with repeated measures was used to analyze group differences in the training trial. The results of the probe trial and WB were analyzed by one-way ANOVA, and the least significant difference (LSD) pairwise comparison method was used among groups. Data are expressed as the means ± SD. Statistical significance was set at *p* < 0.05, and high statistical significance was set at *p* < 0.01.

## Results

### EA Intervention Ameliorates Cognitive Impairment in APP/PS1 Mice

**FIGURE 2:**
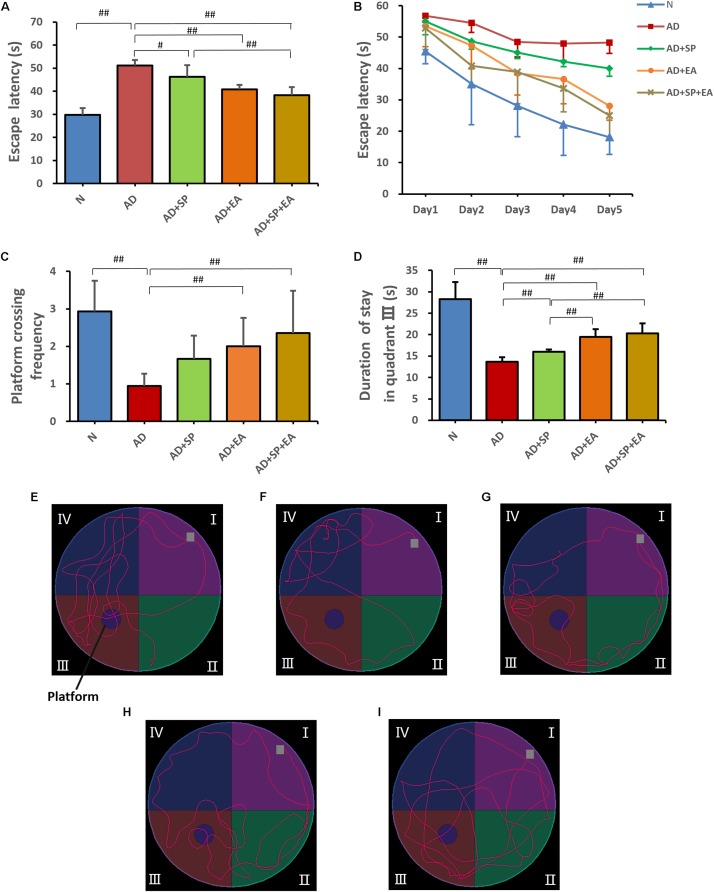
Morris Water Maze test results after intervention (*n* = 10, mean ± SD). **(A)** Comparison of the average escape latency of all groups in training trials. **(B)** Trends of the escape latency of each group in training trials. **(C)** Platform crossing frequency of each group. **(D)** Duration of stay in quadrant III of each group. **(E–I)** Representative probe traces of each group. The water entry points are indicated by gray squares. *n* = 10 per group. ^#^*p* < 0.05; ^##^*p* < 0.01.

In the MWM training trials, the mice in every group showed a downward trend in escape latency from day 1 to day 5 (Figures 2A,B). However, compared with the N group, the AD group showed worse spatial learning performance over all training sessions (*P* < 0.01). Compared with the escape latency of the AD group, the escape latency of the AD + EA and AD + SP + EA groups was lower and significantly lower on days 4 and 5 (*P* < 0.01). Compared with the AD + SP group, the AD + EA and AD + SP + EA groups also showed significantly lower escape latency on day 5 (*P* < 0.01).

In the MWM probe trial on day 6, platform crossing frequency and time spent in quadrant III were tested (Figures 2C,D). A higher platform crossing frequency and greater amount of time spent in quadrant III indicate a higher level of memory maintenance. The platform crossing frequency in the AD group was significantly lower than that in the N group (*P* < 0.01). However, compared with the AD group, the AD + EA and AD + SP + EA groups showed a significantly greater number of platform crossings (*P* < 0.01). Furthermore, the AD + EA and AD + SP + EA groups spent more time in quadrant III than the AD + SP group (*P* < 0.01). Figures 2E–I shows the representative strategies for searching for the platform of each group. The AD group showed an edge search strategy, suggesting that 7-month APP/PS1 mice displayed obvious impairment in learning and memory. The N group showed a search strategy that was similar to that of the AD + SP, AD + EA and AD + SP + EA groups.

### EA Intervention Lowers the Burden of APP in the Hippocampus of APP/PS1 Mice

**FIGURE 3:**
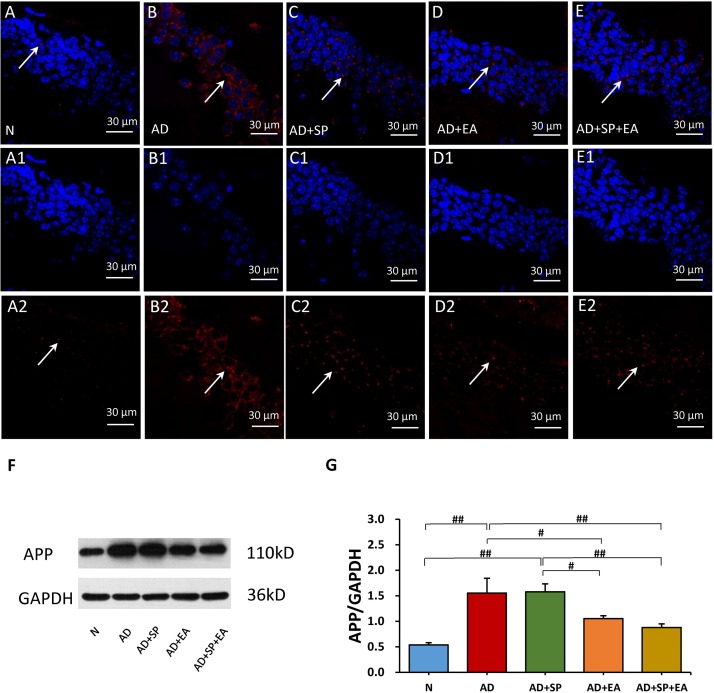
Comparison of APP expression in the hippocampus of APP/PS1 mice in each group. **(A–E)** Comparison of APP expression using immunofluorescence. **(A1–E2)** Comparison of APP expression in the square frame of A-E in detail. APP: red; DAPI: blue; White arrows indicate positive expression of APP. **(F,G)** Comparison of APP expression using WB. Data are presented as the means ± SD, WB: *n* = 7 per group, immunofluorescence: *n* = 3 per group. ^#^*p* < 0.05; ^##^*p* < 0.01.

We next evaluated the distribution and accumulation of APP in the mouse brain hippocampus by immunofluorescence and WB. Immunofluorescence showed the expression of APP in the hippocampus, with obvious higher expression in the AD group (Figures 3A–A2,B–B2) that was decreased in the AD + SP, AD + EA, and AD + SP + EA groups (Figures 3C–C2,D–D2,E–E2). WB results showed notably higher accumulation of APP in the AD and AD + SP groups compared to that in the N group (*P* < 0.01), while the AD + EA and AD + SP + EA groups showed lower expression of APP than the AD group (*P* < 0.05 and *P* < 0.01). Furthermore, the AD + EA and AD + SP + EA groups showed lower expression of APP than the AD + SP group (*P* < 0.05 and *P* < 0.01) (Figures 3F,G). These results confirmed the efficacy of the EA intervention in decreasing the deposition of APP.

### EA Intervention Inhibits JNK Signaling Pathway Activation in the Hippocampus of APP/PS1 Mice

**FIGURE 4:**
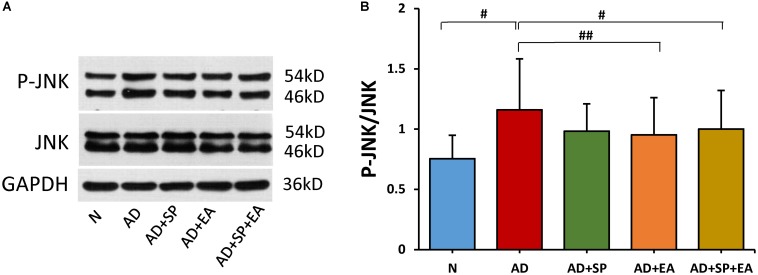
Comparison of the expression of JNK in the hippocampus of APP/PS1 mice using WB. **(A)** The relative expression of hippocampal phosphorylated JNKs and total JNKs in APP/PS1 mice. **(B)** The ratio of p-JNK expression level to the total JNK level in the hippocampus of APP/PS1 mice. Data are presented as the means ± SD, WB: *n* = 7 per group. ^#^*p* < 0.05; ^##^*p* < 0.01.

We hypothesized that the decrease in APP deposition is related to the JNK signal transduction pathway. To test this possibility, we first detected the expression of JNK by WB. The result shows that the JNK phosphorylation in AD group was increased significantly when compared to N group (*P* < 0.05). Here, decline trend of P-JNK phosphorylation were also seen after SP600125 intervention. However, as compared to AD group, EA resulted in significant reduction in P-JNK phosphorylation in the AD + EA and AD + SP + EA groups (*P* < 0.05 and *P* < 0.01). The results supported that EA mainly inhibited activation of JNK in AD (Figures 4A,B).

**FIGURE 5:**
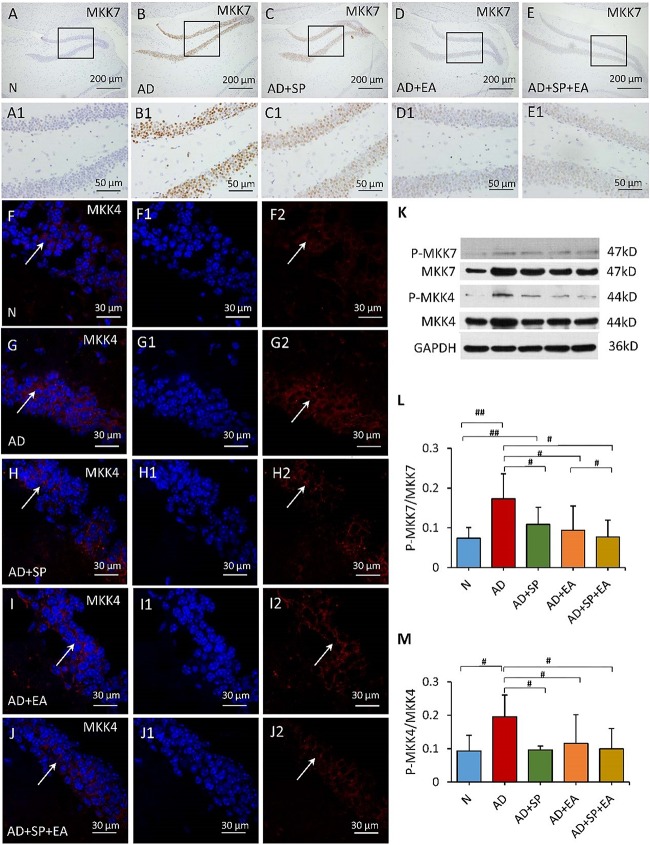
Comparison of MKK7 and MKK4 expression in the hippocampus of APP/PS1 mice in each group. **(A–E)** Comparison of MKK7 expression using immunohistochemistry. **(A1–E1)** Comparison of MKK7 expression in the square frame of **(A–E)** in detail. **(F–F2,G–G2,H–H2,I–I2,J–J2)** Comparison of MKK4 expression using immunofluorescence. MKK4: red; DAPI: blue; White arrows indicate positive expression of MKK4. **(K)** The relative expression of hippocampal phosphorylated MKK7 and MKK4 and total MKK7 and MKK4 in APP/PS1 mice. **(L,M)** The ratio of p-MKK7 and p-MKK4 expression level to the total MKK7 and MKK4 level in the hippocampus of APP/PS1 mice. Data are presented as the means ± SD, WB: *n* = 7 per group, immunofluorescence: *n* = 3 per group. ^#^*p* < 0.05; ^##^*p* < 0.01.

**FIGURE 6:**
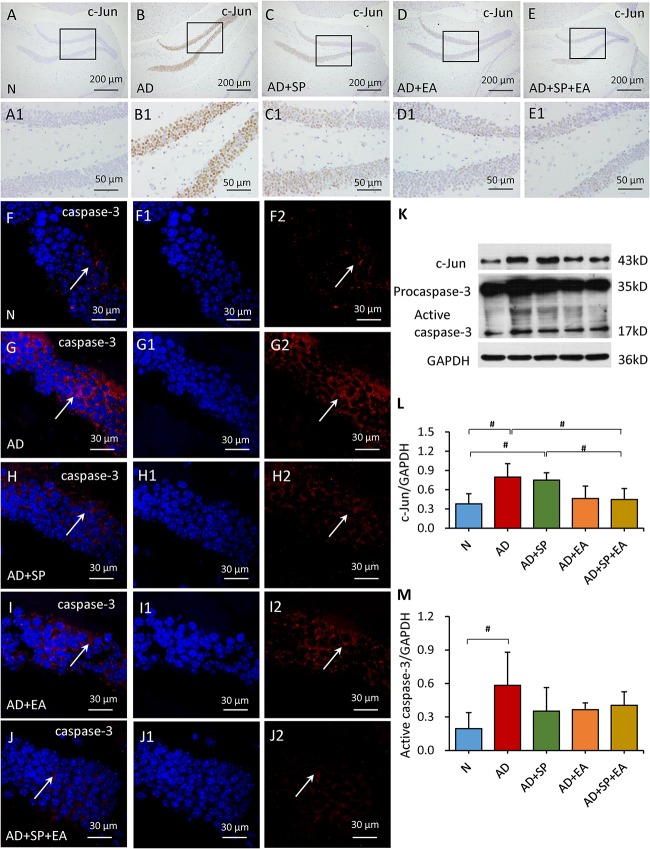
Comparison of the expression of c-Jun and caspase-3 in the hippocampus of APP/PS1 mice in each group. **(A–E)** Comparison of c-Jun expression using immunohistochemistry. **(A1–E1)** Comparison of c-Jun expression in the square frame of **(A–E)** in detail. **(F–F2,G–G2,H–H2,I–I2,J–J2)** Comparison of caspase-3 expression using immunofluorescence. Caspase-3: red; DAPI: blue; White arrows indicate positive expression of MKK4. **(K–M)** Comparison of the expression of c-Jun and caspase-3 using WB. Data are presented as the means ± SD, WB: *n* = 7 per group, immunofluorescence: *n* = 3 per group. ^#^*p* < 0.05.

To further confirm the linkage between APP and the JNK signal transduction pathway, we detected the expression of MKK4, MKK7, c-Jun and caspase-3 in the mouse brain hippocampus by immunofluorescence, immunohistochemistry and WB. In the immunohistochemistry and immunofluorescence analyses, MKK4, MKK7, c-Jun and caspase-3 expression was obviously higher in the AD group than in the N group, but this expression decreased after the intervention in the AD + SP, AD + EA and AD + SP + EA groups (Figures 5A–J2, 6A–J2). WB results showed notably higher expression of p-MKK4, p-MKK7, c-Jun and caspase-3 in the AD group compared to that in the N group (*P* < 0.05 or *P* < 0.01) (Figures 5K–M, 6K–M). Compared to the expression in the AD group, the expression of p-MKK7, c-Jun in the AD + EA and AD + SP + EA groups was significantly lower (*P* < 0.05 or *P* < 0.01) (Figures 5K,L, 6K,L). Furthermore, the AD + SP + EA group showed significantly lower expression of MKK7 and c-Jun than the AD + SP group. The results suggested that EA exerts its effects mainly by regulating MKK7 and c-Jun in the JNK pathway.

## Discussion

In this study, EA treatment improved the learning and memory ability of AD model APP/PS1 mice, which is consistent with a previous study. EA also inhibited JNK activation and APP accumulation in APP/PS1 mice. The data suggested that EA treatment may effectively improve cognitive impairments in AD, at least partially by inhibiting the JNK signaling pathway.

AD is a multifaceted neurodegenerative disease that is clinically characterized by progressive deterioration of cognitive functions. Increasing evidence points to a pivotal role of the JNK signaling pathway in the development of AD. The JNKs, known as stress-activated protein kinases (SAPKs), belongs to the family of mitogen-activated protein kinases (MAPKs). JNKs are a family of multifunctional signaling molecules that are activated in response to a wide range of cellular stresses and are involved in the regulation of cell proliferation, differentiation and apoptosis (Dhanasekaran and Reddy, 2008). SP600125 is an anthrapyrazolone inhibitor that binds to JNK to inhibit phosphorylation and subsequently blocks the functional activation of JNK. Therefore, the JNK-specific inhibitor SP600125 may potentially reduce JNK activity to prevent neuronal degeneration (Sharma et al., 2010). In the present study, both EA and SP600125 ameliorated cognitive deficits by improving spatial learning and memory. More interestingly, the protective effect was more remarkable when the two interventions were combined. The JNK-specific inhibitor SP600125 may potentially reduce JNK activity to prevent neuronal degeneration (Sclip et al., 2011; Sherrin et al., 2011).

APP plays a pivotal role in the pathophysiology of AD. APP metabolism drives other pathological changes, including the accumulation of Aβ. The accumulation of Aβ plays the most important role in the pathogenesis of AD. The Aβ stimulated microglial inflammatory responses engage MAPK pathways in AD. The major component of AD-associated amyloid-beta load is the 39- to 42-residue-long Aβ peptide. In previous study, our team found that the EA treatment was effective in decreasing the Aβ in AD mice, including Aβ_1__–__40_ and Aβ_1__–__42_ in hippocampus, cortex and serum (Wang X. et al., 2016). By reducing APP, it can indirectly reduce the production of Aβ and alleviate the accumulation of Aβ. Thus, therapy that targets APP metabolism is considered to be an effective approach to treat AD. According to our previous studies, EA treatment reduced APP expression by regulating β-site APP–cleaving enzyme 1 (BACE1) in APP/PS1 mice and regulated protein kinase A (PKA) and its associated substrates to change memory and learning abilities (Tang et al., 2019). In the present study, APP expression in the AD + SP group was not significantly different from that in the AD group, but APP expression in the AD + EA group was significantly reduced. Some investigations showed Aβ activated the expression of BACE1 through the JNK pathway (Guglielmotto et al., 2011). BACE1 is an β-secretase, which cleaves the ectodomain of APP. However, the inhibition of JNK activation by SP600125 might be decrease the BACE1, resulting in APP accumulation. In addition, SP600125 was injected intraperitoneally in this study, the effect of SP600125 might be weakened by the blood-brain barrier. These results suggest that EA could promote the curative effect of SP600125. More importantly, the effects of EA might not be entirely mediated through the JNK signaling pathway and that EA effects multiple AD-associated pathways (Colombo et al., 2009; Guglielmotto et al., 2011). Acupuncture may enhance the cognitive function-improving effect of SP600125 in the treatment of AD (Mazzitelli et al., 2011; Zhou et al., 2015).

Numerous studies have reported an increase in the abnormal activation of JNK in both transgenic AD mouse models and AD patients. Indeed, aberrant activation of JNK has been implicated in the pathogenesis of AD (Lagalwar et al., 2006). We also found that the level of P-JNK was significantly higher in APP/PS1 mice. To date, three JNKs have been identified in cells, namely, JNK1, JNK2 and JNK3 (Sherrin et al., 2011; Petrov et al., 2016). Studies have shown unequivocal evidence that JNK1 and JNK2 are involved in apoptotic signaling (Busquets et al., 2019). Studies have also shown that the transcriptional activity promoted by Aβ_1__–__42_ on BACE1 is transmitted by the activation of the JNK pathway. JNK activation regulates the phosphorylation of APP, leading to modulation of Aβ levels (Colombo et al., 2009). In this study, the P-JNK levels declined by varying degrees after the interventions. Notably, compared with SP600125, EA treatment has an significantly inhibitory effect on the activation of JNK, which may be due to the incomplete blocking of the blocker. However, EA works in many ways, which is also the advantage of EA, so its effect is not affected by blockers.

MKK4 and MKK7, two MAPK kinases, are key upstream factors of the JNK signaling pathway located in the cytoplasm. Both MKK4 and MKK7 are capable of dual phosphorylation of JNK and activation of JNK (Nakagawa et al., 2010). MKK4 can activate JNKs as well as p38MAPKs, whereas MKK7 specifically activates JNKs (Haeusgen et al., 2011). In the present study, the expression of both p-MKK4 and p-MKK7 was greatly increased in APP/PS1 mice, and this increase was responsible for the high level of P-JNK. MKK4 has been reported to be primarily activated by environmental stresses, whereas MKK7 is primarily activated by cytokines. In addition, MKK7 is the only MKK containing three motifs within its regulatory domain (Haeusgen et al., 2010; Kragelj et al., 2015; Wang S. et al., 2016). The three interventions examined here led to substantially reduced MKK4 and MKK7 expression. Notably, EA in APP/PS1 mice significantly reduced the level of MKK7, indicating that EA treatment inhibits JNK through acting on MMK7 expression (Ho et al., 2006).

The JNK signaling pathway involves phosphorylation of c-Jun. JNK is a kinase of c-Jun, a specific transcription factor that promotes c-Jun phosphorylation and activates caspase cascade reactions to initiate cell apoptosis (Nishina et al., 2004). Both c-Jun and caspase are key downstream factors of the JNK signaling pathway (Kim et al., 2005). Studies have confirmed that activation of caspase-3 occurs in Aβ-induced neuronal apoptosis (Chu et al., 2015; Chang et al., 2016). The role of JNK signaling pathway in cell stress response is complex and diverse. Its activation promotes the occurrence of cell apoptosis, and its mechanism is related to the induction of FasL and TNFR1 expression and the activation of caspases family. According to some studies, JNK may be the upstream regulatory molecule of caspase-dependent apoptosis signal transduction pathway, and caspases act as the effector of apoptosis in the downstream of JNK (Choi et al., 1999). On the other hand, caspases can induce JNK phosphorylation by activating upstream protein kinases in the MAPKs pathway, leading to apoptosis (Newhouse et al., 2004). In addition, Caspase-3 has been shown to cleave APP giving rise to amyloidogenic fragments including Aβ (Gervais et al., 1999). In this study, the expression of both c-Jun and caspase-3 was significantly elevated in APP/PS1 mice, and this increase in expression was due to the high level of JNK phosphorylation and activation. After EA, the level of c-Jun in APP/PS1 mice was greatly reduced. Consistently, previous reports have shown that JNK2 has the highest affinity for c-Jun, as it contains the putative loop region that interacts with the JNK docking site on c-Jun. Furthermore, the level of caspase-3 showed a downward trend with EA treatment. Therefore, we demonstrate that JNK signaling pathway could be involved in anti-apoptotic effect of APP/PS1 mice. The results of this study highlighted that EA combined with the SP-induced (SP600125)could regulate the JNK signaling pathway and inhibit the production of caspase-3 of APP/PS1 mice, indicating the beneficial role of EA in neuron functional reconstruction and reducing the apoptosis. Collectively, our findings, along with previous reports, demonstrate that the inhibition of JNK activation by EA may occur through multiple targets.

**FIGURE 7:**
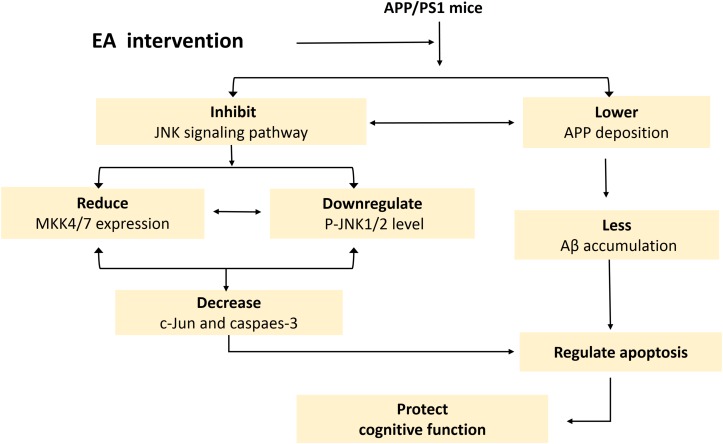
Schematic of the mechanism by which EA intervention protects cognition in APP/PS1 mice. This schematic is derived from the results of this experiment combined with the results of our previous experiments.

In summary, this study provides essential preclinical evidence suggesting that EA reverses cognitive deficits and substantially lowers the burden of APP in AD model APP/PS1 mice, at least partially by inhibiting the JNK signaling pathway and regulating apoptosis signals (Yarza et al., 2016) (Figure 7). In addition, our findings indicated a moderate synergy between the EA and SP600125 interventions in APP/PS1 mice. Collectively, these data suggest that EA is effective at treating AD and may enhance the effect of drugs. Therefore, EA offers an effective alternative therapeutic approach for AD.

## Data Availability Statement

The raw data supporting the conclusions of this article will be made available by the authors, without undue reservation, to any qualified researcher.

## Ethics Statement

All experimental procedures were in accordance with the Guide lines for the Care and Use of Laboratory Animals of the Ministry of Science and Technology of the People’s Republic of China, and the Experimental Animal Research Ethics Committee of Beijing University of Chinese Medicine approved the study protocol.

## Author Contributions

YT and AX: experimental design, data analysis, and manuscript preparation. SS: data collection. YZ, BX, and ZL: experimental design.

## Conflict of Interest

The authors declare that the research was conducted in the absence of any commercial or financial relationships that could be construed as a potential conflict of interest.
